# Examining How Black Women Medical Students Rate Their Experiences with Medical School Mistreatment on the Aamc Graduate Questionnaire

**DOI:** 10.5334/pme.1188

**Published:** 2024-04-29

**Authors:** Sacha Sharp, Christen Priddie, Ashley H. Clarke

**Affiliations:** 1 Department of Medicine at Indiana University School of Medicine, Indianapolis, Indiana, USA; 2 Center for Postsecondary Research, Indiana University Bloomington, Bloomington, Indiana, USA; 3 Johns Hopkins Carey Business School, Baltimore, Maryland, USA

## Abstract

**Introduction:**

Few researchers have examined how medical student mistreatment varies by race/ethnicity and gender, specifically highlighting Black women’s experiences. Moreover, researchers often fail to use theoretical frameworks when examining the experiences of minoritized populations. The purpose of this study was to examine the frequency of mistreatment US Black women medical students experience and how this compared to other students underrepresented in medicine (URiM) using intersectionality as a theoretical framework.

**Methods:**

We used the Association of American Medical Colleges Graduate Questionnaire (GQ) as the data source for examining descriptive statistics and frequencies. We examined differences between US Black women (N = 2,537) and other URiM students (N = 7,863) with Mann-Whitney U tests.

**Results:**

The results from this study highlighted that most Black women medical students did not experience mistreatment, yet a higher proportion of these trainees reported experiencing gendered (χ^2^(1) = 28.59, p < .01) and racially/ethnically (χ^2^(1) = 2935.15, p < .01) offensive remarks at higher frequency than their URiM counterparts. We also found US Black women medical students infrequently (27.3%) reported mistreatment from a lack of confidence for advocacy on their behalf, fear of reprisal, and seeing the incident as insignificant.

**Discussion:**

A paucity of research exists on Black women medical students and even less using relevant theoretical frameworks such as intersectionality. Failure to extract Black women’s experiences exacerbates alienation, invisibility, and inappropriate attention to their mistreatment.

## Introduction

Medical student mistreatment remains a challenge and a detriment to academic success [1,2]. Mistreatment in medical school includes discrimination, verbal abuse, public humiliation, assaults and harassment, and has been associated with burnout and attrition [1]. In various instances of mistreatment, medical students report abuse categorized as rude, demeaning, bullying, intimidation, poor anger management, and aggressive interrogation [3]. Further, medical students report incidents of medical mistreatment as “denied opportunities, lower evaluations or grades, and offensive names and remarks predicated on gender, race/ethnicity, and/ or sexual orientation [4 (p.705)].” Public humiliation takes place when a preceptor or attending knowingly asks questions that the medical student is sure to respond incorrectly [5]. These incidents of medical student mistreatment are perpetuated throughout medical education where educators and faculty teach and model behaviors suggestive of incivility within the medical education ecosystem [3].

Medical student mistreatment persists in medical education environments because of the disparate power dynamics that medical students often experience with nurses, residents, fellows, and attendings being in hierarchical positions within hospital teams [2]. Because medical students are at the bottom of the hierarchy, they tend to hold the least amount of agency when responding to difficult situations. Additionally, the constant transition of medical students to new teams diminishes the ability and capacity for medical students to establish themselves as knowledgeable and reliable team members. The excessive workload of needing to acclimate to the new environment, navigate patient encounters, contribute appropriate medical knowledge, complete clerkship requirements, and study for exams create an additional strain on medical students’ mental health [6,7]. This additional strain makes them more susceptible to mistreatment [8]. Furthermore, the patriarchal hegemony that dominates medicine dictates that minoritized groups often experience the most harm in terms of mistreatment. In this article, we explore how patriarchal hegemony impacts the mistreatment of minoritized medical students using intersectionality as an analytical tool for capturing experiences.

Although several researchers have confirmed the prevalence of medical student mistreatment in medical school [9,10], few have highlighted how it varies across race and gender [1]. Moreover, there is little to no research that specifically explores medical student mistreatment among Black women in comparison to their underrepresented peers. In a recent narrative review [11], researchers explored literature that used intersectionality as a framework to underscore how Black women medical students are rendered invisible in medical education and literature. Similarly, scholars have suggested using intersectionality to explore Black women’s invisibility across other disciplines, such as education, sociology, and science, technology, engineering, and math (STEM) [12,13].

### Importance of Disaggregating for Black Women

Within medical education, students of color report experiencing mistreatment at higher rates than their white student counterparts [14]. Researchers suggest that the mistreatment among medical students of color is often characterized in their performance evaluations where they are initially presumed to be less intelligent than their white peers [15]. In addition to the pressures and stressors of medical school, students of color are reminded of their racial, ethnic, and gender identity, and experience discrimination and other inequities merely based on their minoritized status [15]. This is significant for Black women who exist at the intersections of multiple marginalized identities. From an intersectional perspective, Black women face multiple forms of discrimination lending to stress related concerns [16].

According to a US study conducted by Hill et al., [1] women who identify as underrepresented in medicine (URiM) report the highest levels of racial and ethnic discrimination. This study was performed using 2016–2017 data from the Association of American Medical Colleges (AAMC), which unfortunately did not disaggregate for categories of URiM status. That said, the results of this research study suggest that Black women could be experiencing high levels of mistreatment. Therefore, it is necessary to conduct research that can isolate this demographic to better understand their experiences and the levels of mistreatment US Black women encounter in medical school.

When researchers focus on Black women, it means they accurately represent and report the possible vulnerabilities Black women experience as they engage the medical profession. Living at the intersection of racism and sexism, Black women have unique experiences that cannot be captured if researchers continue to aggregate data, even with a focus on URiM. It is well documented that a diverse workforce will lead to greater health equity [6,17,18]. Therefore, focusing on the needs of Black women medical students can lead to greater health outcomes for all. The purpose of this paper was to present results from the AAMC Graduate Questionnaire (GQ) about mistreatment among US Black women medical students. In the study, we asked the following research question:

At what frequency do US Black women medical students rate their experiences with mistreatment in medical school compared to their (URiM) counterparts?

## Theoretical Framework

For this research, we used intersectionality as a theoretical framework to highlight Black women’s nuanced experiences during their medical education. Crenshaw [19] argued that intersectionality removes the single-axis framework for understanding identity and the experiences associated with one’s identity. Instead, intersectionality names how discrimination and oppression are compounded from the multiple marginalized identities that people hold [19]. Intersectionality may be interpreted as a three-dimensional tool for critically analyzing discrimination and oppression. The three dimensions of intersectionality are structural, political, and representational. For the scope of this study, we focus primarily on structural intersectionality.

Structural intersectionality may be used to understand how the single-axis identity renders Black women invisible. Black women’s experiences are absent from research as the focus of social inequity discourse captures one identity, namely race or gender. This single-axis, or race-only versus gender-only, approach to research most often presents data on Black men or white women as these two groups are the “minoritized default [11 (p.3)].” Since Black women experiences are at the intersection of race and gender, structural intersectionality offers an analytical viewpoint to understand Black women perspectives more fully.

Using intersectionality as an analytical tool reveals how Black women’s race and gender make them invisible in most spaces, including academic research [20,21]. This notion of invisibility further underscored our argument for disaggregating data so that Black women’s multidimensional experiences are present in literature, and thus, leads to more ideal environments for thriving in their education. For this study, we are guided by intersectionality as the theoretical framework to analyze the GQ data on US Black women’s experiences in medical school. We also offer critique of this data source for consistently reporting information on URiM students in aggregated ways. As noted, we used structural intersectionality as a lens to understand US Black women medical students’ experiences.

## Method

### Positionality

The research team was made up of three Black women who each hold a PhD in higher education. We serve as an assistant professor, an assistant research scientist, and a senior administrator. We each have expertise in conducting research with minoritized populations that enroll in STEM programs and professional schools. Moreover, because we hold intersectional identities, we have the unique perspective that required us to explore the research question using intersectionality as a framework.

### Data Source

The data used for this study is secondary data from the GQ. A data licensing agreement was established between AAMC and the lead author’s home institution regarding the ethical use of this data. The data licensing agreement also outlined how data is to be explicitly used for the expressed purpose of research on the experiences of Black women medical students. The GQ survey is administered annually to over 20,000 fourth year US medical students and has them rate their level of satisfaction with clinical experiences, experience with residency readiness, career intentions, and indicate whether they have experienced negative behaviors they may experience while in medical school [22]. Each year, the GQ is administered from February to June to medical students from US medical degree granting institutions who are expecting to graduate by the end of the academic year. Participation in the GQ is voluntary and since 2018, the GQ has received over 16,000 medical student responses each administration year. The GQ does not release any personal information of students when providing data for research purposes and has undergone the necessary IRB approvals through the American Institute of Research. More information about the GQ can be found at https://www.aamc.org/data-reports/students-residents/report/graduation-questionnaire-gq [22].

### Measures

We received a data request approval by the GQ to specifically study deidentified data of Black women medical school experiences in the United States between 2018 and 2021. Across 2018–2021, 66,121 medical school graduates responded to the GQ. This study specifically focuses on a secondary data analysis of 2018–2021 GQ data regarding negative behavior experiences and reporting experiences for 2,840 Black women medical graduates and 8,503 URiM medical graduates.

#### Mistreatment

Mistreatment was measured using seven selected items asking medical students about the frequency at which they experienced public humiliation, denial of opportunities based on race and gender, offensive sexist and/or racist remarks, and receiving lower evaluations based on race and gender. The four-point Likert scale response option ranged from 1 (Never) to 4 (Frequently).

#### Reporting Behaviors

Reporting behaviors were measured through two items. The first item was a yes/no question asking medical students if they reported behaviors to a faculty member or administrator. This item only included responses from those who indicated they experienced mistreatment ‘Once’, ‘Occasionally’, or ‘Frequently’ on the mistreatment items noted above and in Table 1. The second item asked medical students to indicate why they did not report mistreatment through a ‘check all that apply’ response option. Two additional items asked whether respondents witnessed other students being mistreated and whether they reported witnessed behaviors using a yes/no response option.

**Table 1:** Frequency and Chi-Square Information for U.S. Black Women and URiM Medical Students’ Mistreatment Experiences.


		BLACK WOMEN (N = 2,537)	URiMs (N = 7,863)	CHI-SQUARE (p-VALUE)*

**Publicly humiliated**	Never	68%	74.6%	29.63 (<.01)

	Once	17.7%	15%	

	Occasionally	12.7%	9.6%	

	Frequently	1.7%	0.8%	

**Denied opportunities based on gender**	Never	93.8%	92.9%	1.07(.30)

	Once	2.8%	3.3%	

	Occasionally	3%	3.3%	

	Frequently	0.4%	0.5%	

**Been objected to offensive sexist remarks/names**	Never	77%	83.7%	28.59(<.01)

	Once	10.8%	7.6%	

	Occasionally	11.1%	7.7%	

	Frequently	1.1%	1.0%	

**Received lower evaluations based on gender**	Never	89.7%	92.5%	23.87(<.01)

	Once	5.5%	4.7%	

	Occasionally	4.3%	2.3%	

	Frequently	0.5%	0.5%	

**Denied opportunities based on race/ethnicity**	Never	85.6%	94.7%	165.02(<.01)

	Once	5.5%	2.4%	

	Occasionally	7.2%	2.3%	

	Frequently	1.7%	0.7%	

**Been objected to racially/ethnically offensive remarks/names**	Never	70.3%	86.3%	235.15(<.01)

	Once	12.9%	7.0%	

	Occasionally	15%	6.0%	

	Frequently	1.7%	0.6%	

**Received lower evaluations based on race/ethnicity**	Never	80.9%	94.8%	337.07(<.01)

	Once	8.1%	2.9%	

	Occasionally	8.5%	1.8%	

	Frequently	2.5%	0.5%	


* For chi-squared tests, response options were dichotomized to where Never and Once were combined to represent ‘Infrequently’ and Occasionally and Frequently were combined to represent ‘Frequently’.

#### Quality of Medical Education

Satisfaction with quality of medical education was measured with one item asking respondents about their overall satisfaction with the quality of their medical education, with a five-point Likert scale response option ranging from 1 (Strongly disagree) to 5 (Strongly agree). We understand that the term ‘diversity’ can be interpreted differently by our Black women medical student sample than other medical students, especially as Black women may see themselves as the diversity representation. However, we felt the inclusion of this item was important as it is one of the few GQ items that discusses diversity within the medical education environment.

#### Revisit Career Choice

Revisiting career choice was measured with one item asking respondents whether they would choose to attend medical school again if they could revisit their career choice, with a five-point Likert scale ranging from 1 (No) to 5 (Yes).

#### Skill Enhancement Through Medical School Diversity

Diversity within medical school was measured with one item asking respondents about their agreement with thoughts that diversity within their medical school enhanced their training and skills to work with people from different backgrounds. The response options were a five-point Likert scale, ranging from 1 (Strongly disagree) to 5 (Strongly agree).

##### Demographic Information

As noted above, only data for US Black women were used in subsequent analysis. Race/ethnicity was measured through one ‘check all that apply’ item asking respondents how they self-identify. URiM in subsequent analysis were combined to include American Indian or Alaska Native, Hispanic, Latino or of Spanish origin, Native Hawaiian or Other Pacific Islander, and Other. Our definition of URiM is consistent with the AAMC definition, which means individuals from racial and ethnic backgrounds who are underrepresented as compared to their representation in the general population [22]. That said, the GQ generally excludes the ‘Other’ category from URiM analysis. However, for this study we include “Other” to capture individuals that may be from underrepresented categories, but who do not know how they fit within the US context of race and ethnicity. Additionally, Black women who also identify as another race are included in this analysis. Therefore, if a woman checks Black and Asian, she is included as part of the analysis for Black women experiences of mistreatment. Sex was measured with one item asking whether respondents identified as ‘Male’ or ‘Female’. Only respondents who selected ‘Black or African American’ and ‘Female’ were categorized as Black women in analyses.

### Statistical Analysis

All analyses were determined and performed once we received the secondary data to assess variable type. Decisions to examine descriptive statistics and frequencies were made a priori to data analysis, while the chi-squared tests, Mann-Whitney U tests and Spearman’s rank correlations were determined to be appropriate after secondary data structure was understood. Using Statistical Package for the Social Sciences (SPSS) version 29.0.1.0, we first examined frequencies and chi-squares to answer our research question about Black women medical students’ mistreatment in medical school in relation to their URiM counterparts. We then conducted a series of non-parametric Mann-Whitney U tests to explore how Black women medical students differed in mistreatment experiences compared to other identified URiMs due to the ordinal nature of data. Lastly, due to the ordinal nature of the data, we conducted Spearman’s rank correlations to assess relationships between satisfaction with quality of medical education, revisiting career choice, skill enhancement through medical school diversity, and mistreatment experiences for Black women medical graduates.

## Results

### Respondent Information

Between 2018 and 2021, 2,840 US Black women medical students completed the GQ. The number of Black women respondents increased each year, with 597 Black women in 2018 and 765 in 2021. Most of the Black women medical student respondents were 29–39 years old (46%), single (75%), and had zero dependents (82%). After accounting for missing responses through pairwise deletion, subsequent analyses included responses from 2,840 Black women and 8,503 other URiMs.

### Mistreatment Experiences

We examined frequencies and chi-squared tests to understand the frequency at which US Black women medical students experienced various types of mistreatment in relation to their URiM counterparts (Table 1). It is important to note that for the seven mistreatment items, most respondents for both groups reported never experiencing mistreatment. Therefore, chi-square tests were conducted to examine associations between minoritized group identification and mistreatment experiences. For all chi-square tests, mistreatment response options were dichotomized to where Never and Once were combined to become an ‘Infrequently’ category and Occasionally and Frequently were combined to become a ‘Frequently’ category.

There was a statistically significant association between experiencing public humiliation and minority groups (χ^2^(1) = 29.63, p < .01). Black women medical students were overrepresented in frequently experiencing public humiliation than other URiM medical students. There was a statistically significant association between being objected to offensive sexist remarks (χ^2^(1) = 28.59, p < .01) and receiving lower evaluations based on gender (χ^2^(1) = 23.87, p < .01) for minority groups. Black women medical students were overrepresented in frequently experiences offensive sexist remarks and receiving lower evaluations based on gender than their other URiM medical student counterparts. In terms race/ethnicity, there was a statistically significant association between being denied opportunities based on race/ethnicity (χ^2^(1) = 165.02, p < .01), being objected to racially/ethnically offensive remarks (χ^2^(1) = 235.15, p < .01), and receiving lower evaluations based on race/ethnicity (χ^2^(1) = 337.07, p < .01). Similarly to gender mistreatment, Black women medical students were overrepresented in frequently experiencing denial of opportunities based on race, racially offensive remarks, and receiving lower evaluations based on race/ethnicity. These results provide good insight into the types of mistreatment Black women experience in medical school. However, given how the question for this item was designed, insight into their experiences as Black women through an intersectional lens was absent, a result that became prominent when examining that there were stronger associations between race-based mistreatment items and minority identity than gendered mistreatment items. Structural intersectionality highlights how varying forms of oppression multiply to further marginalize groups and the specific experience of Black women is hidden when survey questions only ask about gender (i.e. “Denied opportunities based on gender”) or race (i.e. “Received lower evaluations based on race/ethnicity”).

### Reporting Mistreatment

Frequencies were examined to assess the reporting behaviors of US Black women and URiM medical students when they experienced mistreatment and why they chose not to report mistreatment (Table 2). Due to the survey item only being administered to respondents who indicated experiencing mistreatment ‘Once’, ‘Occasionally’, or ‘Frequently’ as denoted in Table 1, reporting behaviors are only displayed for 322 US Black women medical students and 812 URiM medical students. Only approximately 27% of Black women medical students who indicated a reporting behavior reported mistreatment to faculty members or administrators to handle their complaints, which is slightly higher than URiMs (24%). For US Black women who chose not to report mistreatment experiences, about 48% of 322 Black women did not report because they “did not think anything would be done about it”, 38%% indicated the “incident did not seem important enough”, and 30% indicated a “fear of reprisal” or retaliation from others. For URiMs who indicated reporting behavior (N = 812), slightly more than half did not report because they indicated “the incident didn’t seem important enough” and about 41% “did not think anything would be done about it”. Additionally, a small percentage of both Black women and URiM medical students are witnessing mistreatment happen to other students but an even smaller percentage are reporting that mistreatment as well. US Black women medical students appear to not report mistreatment at slightly higher percentages than URiMs but it still is concerning that quite a few minoritized medical students are not reporting mistreatment for themselves or their colleagues.

**Table 2:** Percentages of U.S. Black Women and URiM Medical Students Who Selected Reporting Behaviors.


	BLACK WOMEN (N = 322)	URiMs (N = 812)

*Reported behaviors to faculty member or med school admin to handle complaint*	27.3%	24.1%

**Why didn’t you report:		

Reported all incidents	8.7%	7.6%

Incident did not seem important enough	37.6%	51.0%

Resolved issue themselves	18.6%	14.8%

Didn’t think anything would be done about it	47.5%	40.8%

Fear of reprisal	29.8%	31.4%

Didn’t know what to do	14.9%	11.5%

Other	3.4%	7.1%

	**BLACK WOMEN (N = 2,840)**	**URiMs(N = 8,503)**

Witness other students subject to behaviors	6.0%	5.2%

Report witnessed incidents to faculty member or med school admin empowered to handle such complaint	4.5%	3.7%


* Item was only given to respondents who indicated experiencing mistreatment behaviors from Table 1 ‘Once’, ‘Occasionally’, or “Frequently’. ** Percentages only reflect respondents who indicated a reporting behavior from row above and selected one of these options with ‘Check all that apply’ response option. Percentages will not add up to 100%.

### Mistreatment Comparison Between Black Women and Other URiMs

A series of Mann-Whitney U tests were conducted and analyzed to examine whether US Black women and other URiM medical graduates differed on types of mistreatments experienced (Table 3). US Black women experienced statistically significantly more public humiliation than other URiMs (z = –6.87, p < .01), more offensive sexist remarks/names than URiMs (z = –7.66, p < .01), and more racially/ethnically offensive remarks/names than URiMs (z = –18.64, p < .01). We also found that Black women medical students experienced statistically significantly lower evaluations based on race/ethnicity (z = –21.81, p < .01) and gender (z = –4.53, p < .01) more than URiMs and denial of opportunities based on race/ethnicity (z = –15.14, p < .01). For more detailed results see Table 3.

**Table 3:** Mann-Whitney U Results for U.S. Black Women Medical Students’ Mistreatment Compared to Other URiMs.


	BLACK WOMEN	URiMs	

	MEAN RANK (N)	MEAN RANK (N)	z-VALUE

Publicly humiliated	5477.75 (2,537)	5111.04 (7,863)	–6.87*

Denied opportunities based on gender	5162.05 (2,533)	5206.94 (7,863)	–1.49

Been objected to offensive sexist remarks/names	5459.17 (2,530)	5108.61 (7,857)	–7.66*

Received lower evaluations based on gender	5303.48 (2,525)	5155.28 (7,857)	–4.53*

Denied opportunities based on race/ethnicity	5551.28 (2,521)	5076.79 (7,862)	–15.14*

Been objected to racially/ethnically offensive remarks/names	5936.59 (2,527)	4989.47 (7,863)	–18.64*

Received lower evaluations based on race/ethnicity	5738.41 (2,517)	5012.38 (7,859)	–21.81*


* < .01.

### Satisfaction, Diversity, Career Outcomes, and Mistreatment Experiences

Due to the ordinal nature of the data, Spearman’s rank correlations were conducted examining relationships between satisfaction with quality of medical education, revisiting career choice, skill enhancement through medical school diversity, and mistreatment experiences for US Black women medical graduates (Table 4). Satisfaction with quality of education was positively correlated with revisiting career choice and skill enhancement through medical school diversity, while being negatively correlated with experiencing mistreatment. US Black women medical students revisiting their medical career, meaning they would revisit attending medical school again if given the chance, was positively correlated with skill enhancement through medical school diversity and negatively correlated with experiencing mistreatment. Lastly, skill enhancement through medical school diversity was negatively correlated with experiencing mistreatment. Although all correlations were statistically significant, small coefficients indicate that more contextual factors are needed to assess these relationships.

**Table 4:** Spearman Rank Correlations for Quality Satisfaction, Revisiting Career Choice, Medical School Diversity, and Mistreatment for U.S. Black Women Medical Students.


	VARIABLE	1(N)	2(N)	3(N)

1	Medical school satisfaction	–		

2	Revisit career choice	.25* (2491)	–	

3	Skill enhancement due to diversity	.24* (2594)	.12* (2552)	–

4	Experienced mistreatment	–.13* (2763)	–.08* (2559)	–.12* (2670)


* <.01.

### Limitations

There were a series of limitations uncovered during the completion of this study. Some limitations are related to the scope of our secondary data. The GQ has limitations regarding the inclusiveness of demographic items and therefore restricts our ability as researchers to examine our sample beyond gender binaries and beyond a monolithic understanding of Black identity within the United States context. For example, we understand that there are several ethnic identities represented in the Black diaspora that are not captured by the GQ. Further, gender fluidity and orientation are not items captured by this survey, and thus we are unable to further disaggregate data to better understand the nuances of the various groups that may be represented. The GQ is voluntary, therefore this study does not represent the viewpoints of non-respondents. It must also be acknowledged that mistreatment can lead to attrition for some medical students, meaning that because the GQ survey is administered in the fourth year, it does not represent the opinions of students who may have experienced significant instances of mistreatment. In terms of the questions from the GQ, a limitation could be related to the interpretation of the question. Participants and us as researchers may reach different conclusions about what the question is trying to convey. A delimitation is that the GQ is a survey conducted for US medical students. Therefore, the data collection and analysis were produced with society-specific connotations that may not be transferable to other countries. However, information can be gleaned from this study that is relevant to the phenomena of medical school mistreatment in other countries. Lastly, the cross-sectional nature of this research means that only associations can be inferred from the analysis.

## Discussion

According to our results, US Black women medical students generally experience more mistreatment than their URiM peers but have reservations on reporting mistreatment due to little confidence that corrective action will be taken on their behalf. These results are consistent with the sparse literature on medical school mistreatment for Black women, suggesting that medical school mistreatment happens more for Black women than other medical students [1]. The results confirm mistreatment experienced by Black women exist in the form of gendered, racial, and ethnic remarks, public humiliation, and low evaluation scores. The results also highlight how institutions who focus on diversity enhanced training and skill can improve and decrease experiences with mistreatment among US Black women. Conversely, when those diversity enhancements do not exist, the experiences with mistreatment increase for Black women. Taken together, the results suggest institutions that express diversity in their training are in a better position to support Black women, thus mitigating the potential of mistreatment.

According to Dyrbye et al., [17] and Anderson et al., [23] medical students from minoritized backgrounds who experience mistreatment in the form of discrimination are more likely to experience burnout. Our results are consistent with these findings suggesting US Black women who personally experience mistreatment are less likely to revisit being a physician as a career. Moreover, the results highlighted how US Black women are unlikely to revisit being a physician as a career when diversity enhancements were not evident in medical education. These results were consistent with literature that purports poor job satisfaction for physicians who experience mistreatment in the form of discrimination [24].

Lastly, our findings confirmed Anderson et al.’s research [23], which purported medical students who identify with multiple minoritized identities have a higher likelihood of experiencing mistreatment in the form of microaggressions. As we noted, Black women exist at the intersections of both race and gender and have unique experiences that cannot be accounted for when data is viewed from a single access point. That said, our research only accounted for two minoritized identities. Therefore, future research is needed to examine how mistreatment is experienced for individuals who hold multiple minoritized identities beyond race and gender.

## Recommendations

Based on this research, we present several implications for leaders and researchers in medical education. Firstly, it must be acknowledged that research on Black women medical students is an understudied topic. Therefore, it is necessary for more researchers and medical educators to explore the experiences of this demographic to better understand ways of retaining them in the profession. Researchers conducting work in this area tend to group only by race—with a focus on Black men, by gender—with a focus on white women or aggregate all URiM students together [11]. All variations of aggregation miss nuanced differences about the realities of what individuals from multiple minoritized identities experience. By failing to acknowledge and isolate for the intersectional experiences of Black women means to perpetually ignore the gendered racism that Black women face [11]. Furthermore, this lack of consideration is not limited to Black women’s racial and gender identities but may also negatively impact their experiences with the other minoritized identities that Black women hold.

The data collected from the GQ suggested that in addition to experiencing mistreatment, Black women also choose not to report incidents that negatively impacted how they experience medical school. Therefore, we have specific recommendations for medical educators regarding mistreatment reporting. First, institutions of medical education should develop and streamline safe mistreatment reporting structures. Black women medical students who experience mistreatment and do not report for fear of retaliation may exist in environments where they are not protected from the outcomes of their reporting. Anonymous reporting structures may increase the likelihood of Black women reporting mistreatment. We recommend developing reporting structures where Black women students perceive them as safe and discreet throughout the entire investigative process.

Second, all medical students should receive education about what mistreatment is and how to report, to the point that it becomes a regular aspect of the educational culture. This is especially critical for US Black women medical students who experience mistreatment at higher rates than their URiM counterparts. A part of that education should include acknowledgment that it is not the student’s responsibility to decipher if the incident is categorized as mistreatment, and that merely reporting issues of concern is enough.

Our final recommendation is consistent with the results suggesting US Black women experience better satisfaction with medical education when diversity enhanced training and skill are present. At a time when diversity offices in the US are being disbanded and fears about conducting race specific initiatives are at an all-time high, it is important for institutions of medical education to remain steadfast in their commitment to diversity that is reflected in their medical education training.

## Conclusion

This paper contributes to current literature on medical school mistreatment through an intersectional focus on US Black women medical students’ experiences. The results from this study provide an entryway into why solely examining Black women medical students’ experiences is particularly beneficial to researchers and practitioners alike focused on improving Black women’s medical school experiences. Our study also creates space for deeper explorations of medical student mistreatment, which can include examining who is contributing to mistreatment (faculty, staff, peers, etc.), how anti-blackness factors into medical school environments, and better understanding why Black women chose not to report these mistreatment experiences.
